# Secondary prevention of rheumatic heart disease in Ethiopia: a multicenter study

**DOI:** 10.1186/s12872-022-02473-4

**Published:** 2022-02-02

**Authors:** Wubishet Belay, Azene Dessie, Hayat Ahmed, Etsegenet Gedlu, Abinet Mariyo, Abdulkadir Shehibo, Zemene Tigabu, Muktar H. Aliyu, Jonathan Soslow

**Affiliations:** 1 Monroe Carell Jr. Children’s Hospital at Vanderbilt, 2200 Children’s Way 5th Floor, Nashville, TN 37232 USA; 2 Cardiac Center Ethiopia, Addis Ababa, Ethiopia; 3 Black Lion Specialized Referral Hospital, Addis Ababa University College of Medicine and Health Sciences, Addis Ababa, Ethiopia; 4 College of Medicine and Health Sciences, University of Gondar, Gondar, Ethiopia; 5 Vanderbilt Institute for Global Health (VIGH), Nashville, TN USA

**Keywords:** RHD, ARF, Adherence, Prophylaxis

## Abstract

**Background:**

Ethiopia has a high acute rheumatic fever (ARF) and rheumatic heart disease (RHD) prevalence, and to our knowledge, there are no data on the status of secondary prevention in children with RHD. This study describes the status of secondary RHD prevention.

**Methods:**

A multicenter, prospective study was performed on children aged 5–17 years with RHD in Ethiopia. Good adherence was defined as at least 80% completion of benzathine penicillin (BPG) or oral Amoxicillin within the previous year. The primary outcome measure was adherence to prophylaxis, expressed as a proportion. Socio-demographics, severity of RHD, and ARF recurrence were evaluated.

**Results:**

A total of 337 children with a mean age of 12.9 ± 2.6 years were included. The majority (73%) had severe aortic/mitral disease. Participants were on BPG (80%) or Amoxicillin (20%) prophylaxis. Female sex (*P* = 0.04) use of BPG (0.03) and shorter mean duration of prophylaxis in months (48.5 ± 31.5 vs. 60.7 ± 33, respectively, *P* < 0.008) predicted good adherence. Running out of medications (35%), interrupted follow-up (27%), and the COVID-19 pandemic (26%) were the most common reasons for missing prophylaxis. Recurrence of ARF was higher in participants on Amoxicillin compared with BPG (40% vs. 16%, *P* < 0.001) and in those with poor adherence compared with good adherence (36.8% vs. 17.9%, respectively, P = 0.005). Type and duration of prophylaxis (OR 0.5, CI = 0.24, 0.9, *P* = 0.02; OR = 1.1, CI = 1.1, 1.2, *P* = 0.04, respectively), and sex (OR = 1.9, CI = 1.1, 3.4, *P* = 0.03) were independent predictors of poor adherence.

**Conclusion:**

Poor adherence is prevalent in Ethiopian children living with RHD. Amoxicillin is a suboptimal option for prophylaxis as its use is associated with lower adherence and a higher rate of ARF recurrence.

**Supplementary Information:**

The online version contains supplementary material available at 10.1186/s12872-022-02473-4.

## Introduction

Rheumatic heart disease (RHD) is a significant cause of morbidity and mortality in developing nations [1–3]. Ethiopia has one of the highest prevalence of RHD in the world [4, 5], and a substantial proportion of Ethiopian children with chronic RHD present with severe disease [6]. Despite the disease burden, Ethiopia has no national strategy to prevent, control, and treat RHD in children and adolescents.

Secondary prophylaxis protects from recurrent acute rheumatic fever (ARF) and prevents progression to severe forms of RHD [7–10]. The World Health Organization (WHO) defines secondary prevention of ARF as the “continuous administration of antibiotics to patients with a previous episode of ARF or already existing RHD.” A monthly benzathine penicillin (BPG) injection is the recommended prophylactic drug by the WHO and American Heart Association, but oral penicillin V can also be used [8, 11–13].

As surgical care of RHD is unavailable or prohibitively expensive for most patients in Ethiopia, effective prevention is paramount. Findings from studies on adult patients in Ethiopia with RHD indicate that adherence to secondary prophylaxis is variable [14]. Factors affecting adherence in adults include distance from health facilities, lack of finances, and affordability [15, 16]. Although there are no recent data, older adherence studies in children reported similar findings [17, 18].

A well-designed, evidence-based national prevention and treatment strategy is an essential tool to improve care and outcomes. Experience from other countries suggests that an evidence-based national strategy and register-based RHD control programs can improve access for patients with RHD [19–21]. The objectives of this study were to evaluate the level of adherence to RHD prophylaxis and to identify risk factors for poor adherence in Ethiopian children as a first step towards informing effective national prevention, control, and treatment strategies.

## Methods

### Study sites

This multicenter, prospective study was conducted at the University of Gondar, Addis Ababa University, and the Cardiac Center, Ethiopia, from 2019 to 2021. The University of Gondar Hospital is located in northwestern Ethiopia. The hospital has an established pediatric cardiology clinic, which serves as a regional referral center. However, invasive interventions and cardiac surgery are not currently available at this center. Cardiac Center Ethiopia and Black Lion Hospital at Addis Ababa University are located in Addis Ababa, the capital city of Ethiopia. Both centers serve as major referral centers and provide the highest level of care available for RHD patients. Percutaneous catheterization and cardiac surgery are available at Cardiac Center Ethiopia and Black Lion Hospital, although those services can be intermittent and mission-based at times. Most participants in this study came from the central and northern Ethiopian highlands, where more than 90% of the population of Ethiopia lives.

### Recruitment

Institutional research board (Vanderbilt IRB #191171, Addis Ababa IRB 099/20/pedi, Gondar IRB V/P/RCS/05/532/2019), approval was obtained from the participating institutions. Parents/guardians were consented, and assent was obtained from adolescent patients. The consent and assent forms and data collection tools were translated by a certified translation service into the local language and administered to parents/guardians of the children. For parents/guardians who could not read, the consent and assent forms were read by the data collector in the local language. Participants aged 5–17 years with an echocardiogram-confirmed diagnosis of RHD and on prophylaxis for at least 12 months were included during the study period. Patients were only included if they had been on prophylaxis for at least 12 months as it provided enough time to adequately assess adherence. Patients were consecutively enrolled as they presented for follow up. Individuals with congenital heart disease (CHD) were excluded from the study as the presence of CHD complicates the assessment of disease severity.

### Medication adherence

Patients usually get BPG injections or obtain prescriptions of oral Amoxicillin during their pediatric cardiology visits. As per the practice in Ethiopia, patients who can access BPG and live nearby to a health facility that can administer BPG injections were prescribed BPG. Participants who had no access to BPG either due to lack of availability of the drug in their area or lack of trained personnel to administer the injection were prescribed “enough” doses of oral Amoxicillin until the next cardiology clinic visit. Oral Amoxicillin is prescribed for these patients despite not being recommended for secondary prophylaxis due to the unavailability of Penicillin V in Ethiopia. Adherence data were collected with the above considerations in mind.

Adherence was assessed for the 12 months preceding enrollment. Good adherence was defined as 80% completion of the BPG injections (≥ 10 injections per year) for 12 months. For oral amoxicillin, good adherence was defined as completing at least 80% of the prescribed doses each month (≥ 24 tablets per month) in each of 12 months prior to data collection [22].

### Data collection

Data were collected by the pediatric cardiologist or a pediatric cardiology fellow using a questionnaire built in REDCap (Additional file 1). Adherence was primarily determined by interviewing the parents of the participants. The participant/parent or legal guardian were interviewed with structured questions about reasons for missing prophylaxis. Sociodemographic factors such as the participant’s age, sex, address, household size, and family income were captured. The type of prophylaxis (BPG or oral Amoxicillin), frequency of follow-up, and the number of missed prophylaxis doses were recorded. In participants with missed prophylaxis, the reasons for missing the doses were documented. Clinical data on the type and severity of RHD and the number of recurrences of ARF in the past year were recorded. Charts were reviewed for admissions with ARF recurrence and any surgical or catheter-based interventions. All data were entered into a REDCap database.

### Statistical analysis

With an estimated RHD prevalence of 19 per 1000 among children 6–18 years of age, 95% confidence interval, estimated average prevalence of 30% of poor adherence rates, and a margin of error of 5%, we estimated that a representative sample of 330 children would be sufficient. The primary outcome measure was adherence to secondary prophylaxis in RHD patients, expressed as a proportion. Students’ chi-square test and t-test for difference in means were used to assess differences among categorical and continuous variables, respectively. Variables with a significant difference in univariate analysis were selected to build the logistic regression model. Factors independently associated with adherence were determined using multivariate logistic regression, with adherence to prophylaxis classified as a binary variable (yes/no). Odds ratio (OR) and confidence intervals (CI) were reported. A *P* value < 0.05 was considered statistically significant. All analyses were performed using STATA (StataCorp. 2019. Stata Statistical Software: Release 16. College Station, TX: StataCorp LLC.)

## Results

### Demographics

Baseline characteristics of the study population are presented in Table 1. A total of 337 participants were included in this study. The mean age at enrollment was 13 ± 2.6 years, and the mean age at diagnosis of RHD was 8.8 ± 2.7 years. Females accounted for 54% of the cases. Most participants resided in rural areas (62%). The median family size was 6, and 43% of parents had no formal education. Half of all parents (50%) were farmers.

**Table 1:** Sociodemographic characteristics of the study population

Variable		Frequency (%)
Age at enrollment (years)	5–10	34 (10%)
	> 10–17	303 (90%
Sex	Female	182 (54%)
	Male	155 (46%)
Residence	Rural	209 (62%)
	Urban	128 (38%)
Parental level of education	No education	145 (43%)
	Primary school	120 (36%)
	High school	48 (14%)
	College	20 (6%)
	Unknown	4 (1%)
Parental occupation	Farmers	169 (50%)
	Government employees	50 (15%)
	Small scale business/self-employed	76 (22.5%)
	Employed by a private company	42 (12.5%)
Family size	< 6	126 (37%)
	≥ 6	211(63%)
Family income (USD) (mean ± SD)		67 ± 68
Distance from the nearest cardiology clinic ( km)		155 ± 162

### Clinical data

The majority (98%, 330/337) were followed at the nearest pediatric cardiology clinic every 1–6 months. A multivalvular lesion with aortic and mitral valve involvement was seen in 72% (244/337), whereas isolated mitral valve disease was seen in 26% (89/337) of participants. Severe aortic/mitral valve disease was seen in 73% (246/337) of the cases, but only 9% (31/337) had a history of prior valve intervention, either percutaneous (balloon valvuloplasty) or operative.

### Prophylaxis data

The mean duration of prophylaxis was 4.2 ± 2.7 years. BPG (80%) and Amoxicillin (20%) were the only types of prophylaxis used. A total of 54 patients (16%) met the criteria for poor adherence to secondary prophylaxis. Among cases with poor adherence, 30% were on Amoxicillin, and 70% were on BPG (*P* = 0.036). A total of 24.6% of patients on Amoxicillin had poor adherence and 13% on BPG had poor adherence (*P* = 0.04). Predictors of good adherence were female sex (*P* = 0.04), use of BPG (*P* = 0.03), and shorter mean duration of prophylaxis in months (48.5 ± 31.5 vs. 60.7 ± 33, *P* < 0.008) (Table 2). There were no differences in family size, income, disease severity, rural/urban residence, or distance traveled between the two groups. Multivariable logistic regression analysis identified type and duration of prophylaxis (OR 0.5, 95%CI [0.24, 0.9], *P* = 0.02; OR = 1.1, CI [1.1, 1.2], *P* = 0.04, respectively), and sex (OR = 1.9, CI [1.1, 3.4], *P* = 0.03) as independent predictors of poor adherence. (Table 3). The top 3 reported reasons for missing prophylaxis included running out of medicines (n = 19, 35%), interrupted follow-up (n = 15, 27%), and the Severe Acute Respiratory Syndrome (SARS) coronavirus 2019 (COVID-19) pandemic (n = 14, 26%).

**Table 2:** Univariate analysis comparing factors for good and poor adherence in participants with RHD

Variables	Number (n)	Adherence		*P* value
		Good	Poor	
Age at enrollment (mean, CI)	337	12.8 (12.5, 13.1)	13.4 (12.6, 14.0)	0.184
Sex	Females (n = 182)	160 (87%)	22 (12%)	**0.033**
	Males (n = 155)	123 (79%)	32 (21%)	
Family size (mean, CI)	337	6.4 (6.2, 6.7)	5.9 (5.4, 6.5)	0.128
Monthly income in U.S dollars (mean, CI)	337	68.2 ± (60.0, 76.3)	58.9 (42.1, 75.6)	0.352
Residence	Rural (n = 209)	171 (82%)	38 (18%)	0.167
	Urban (n = 128)	112 (88%)	16 (12%)	
Distance from cardiology clinic in kilometers (mean, CI)	337	155.7 (137.0, 174.5)	149 (101.9,196.5)	0.783
Parental education (at least primary school)	Yes (n = 188)	163 (87%)	25 (15%)	0.125
	No (n = 149)	120 (81%)	29 (19%)	
Farmers	Yes (n = 168)	146 (87%)	22 (13%)	0.144
	No (n = 169)	137 (81%)	32 (19%)	
Disease severity	Severe (n = 247)	206 (83%)	41 (17%)	0.630
	Mild to moderate (n = 90)	77 (86%)	13 (14%)	
Prophylaxis	BPG (n = 272)	234 (86%)	38 (14%)	**0.036**
	Amoxicillin (n = 65)	49 (75.4%)	16 (24.6%)	
Duration of prophylaxis (months)	337	48.5 ± 31.5	60.7 ± 33	**0.008**

The bold was to show significance

**Table 3:** Independent predictors of poor adherence to prophylaxis in participants with RHD using multivariate logistic regression

Variables	OR (CI)	*P* value
Sex (females vs. males)	1.9 (1.1, 3.4)	0.039
Prophylaxis (BPG vs. Amoxicillin)	0.5 (0.24, 0.9)	0.027
Prophylaxis duration	1.1 (1.1, 1.2)	0.042

### Recurrence of ARF

Retrospective data on prior recurrences of ARF were obtained in 80% (272/337) of participants. The rest had inadequate documentation. Recurrence of ARF was seen in 20.5% (56/272) of patients over the previous 12 months. Recurrence of ARF was higher in cases on oral Amoxicillin compared to those on BPG (40% vs. 16%, *P* < 0.001), and in those with poor adherence than those with good adherence (36.8% vs. 17.9%, respectively, *P* = 0.005).

## Discussion

Our study demonstrates that Ethiopian children with RHD have a high prevalence of self-reported poor adherence to secondary prophylaxis of RHD. The adherence rate is better in this study compared to adult studies in a similar setting, but comparable studies in children are lacking [14, 15, 23]. Although the median age is higher (24 years) than our study participants, the Global Rheumatic Heart Disease Registry (REMEDY) study showed 78% of their participants had good adherence, similar to our findings [24]. The relatively high recurrence rate in participants with self-reported good adherence (n = 42, 18%) suggests an inherent bias in self-reporting that may underestimate actual adherence. Participants taking oral Amoxicillin had worse adherence to prophylaxis and a higher recurrence rate than those on BPG. This study identified prophylaxis type, duration, and sex as independent predictors of poor adherence in Ethiopia.

Although BPG is the recommended drug to optimize adherence and to reduce the risk of disease progression, a significant number of participants in this study were on oral Amoxicillin for secondary prophylaxis [25]. Oral amoxicillin is recommended for primary prevention of ARF, but there are no studies on its use as secondary prophylaxis. When available, oral penicillin V, not Amoxicillin, is the recommended prophylactic drug. Even then, multiple studies have shown that BPG is superior in preventing the recurrence of ARF. We hypothesize that the improved prevention with BPG is due to better adherence; it is much more difficult to take daily oral medications, while one BPG injection provides 3–4 weeks of protection. In addition, the efficacy of amoxicillin, as compared with penicillin V, is unclear. Recent studies comparing the efficacy of oral penicillin in preventing ARF recurrence are lacking. However, a previous Cochrane review showed a recurrence rate of 16.5% (89/539) in patients taking oral penicillin, which is lower than our cohort on oral amoxicillin [8, 23, 26, 27].

The high rate of severe disease (73%) in the study cohort is also concerning. These results may be due to a referral bias, as participants were drawn from regional referral centers. Although participant demographics are different, the rate of severe disease is about 60% in the REMEDY study, which is lower than our cohort [26]. In addition, females are more affected than males in our study, which is consistent with the findings of the REMEDY study. The high burden of severe disease in our study group illustrates the gravity of RHD in Ethiopia, the need for focused research in this country, and the benefits a national plan to address this epidemic could provide. Most children in this study had never had a cardiac intervention, emphasizing the meager resources available in Ethiopia for patients with severe disease. This finding affirms the critical role of prevention of ARF and RHD in Ethiopia [24, 27, 28].

Although BPG is not expensive, creating the infrastructure to safely administer it requires resources and training. This scenario reflects the poor health systems patients with RHD in Ethiopia face. Kevat et al. showed that adherence is a function of multiple socioeconomic and cultural factors. Although not significant in our study, lack of funds, residing in rural areas, and inconsistent drug supply were reported as additional factors contributing to poor adherence [25, 28, 29].

Health systems and drug supply influence compliance in patients with RHD [29]. For example, our study demonstrated that participants often run out of medications prior to their next visit. These participants are unlikely to refill their prophylaxis medications, as the prescription system in Ethiopia is paper-based. Creating a system for these patients to get their prophylaxis close to home and establishing a sustainable supply chain of medications available at a local health center may help solve this problem. Although there was no difference in family size between those with and without good adherence, the overall cohort had a larger family size than the average in Ethiopia. This is likely secondary the higher risk of streptococcal transmission in larger families [30].

Our study also demonstrates the impact of the COVID-19 pandemic on Ethiopia’s fragile health system. The pandemic has impacted cardiovascular care and contributed to missed follow-up visits. Patients with RHD are sensitive to disruptions in care. Acute healthcare delivery changes brought on by COVID-19 will likely have adverse consequences on these children [31].

Our study has limitations. We collected adherence data based on verbal self-report—participants may therefore over- or underestimate their actual adherence to secondary prophylaxis. Recall bias is also an issue in this type of study. Finally, patients who turn up at regular clinic visits may be more likely to be adherent. Community-based clinical research combined with serum drug level determination or actual pill/injection count by a local community health worker would be a more objective approach to determining adherence. Unfortunately, the infrastructure to measure serum drug levels is currently not available in Ethiopia. Another limitation is the cross-sectional nature of the study, which precludes our ability to make causal inferences.

## Conclusion

Poor adherence and suboptimal secondary prophylaxis are prevalent in Ethiopian children with RHD. Most participants come from rural areas and travel long distances to receive secondary prophylaxis. Severe RHD is rampant, and the recurrence rate is high. General improvement in medication distribution and changes to prescribing practices have the potential to improve adherence. Thus, this study will help inform policies and interventions to enhance RHD prevention programs in Ethiopia and similar settings.

## Supplementary Information

**Additional file 1.** Data collection tool is available as a supplemental file.

## Data Availability

The datasets used and/or analyzed during the current study are available from the corresponding author on reasonable request as long as a data use agreement is signed with Vanderbilt University Medical Center.

## Abbreviations

RHD: Rheumatic heart disease
ARF: Acute rheumatic fever
BPG: Benzathine penicillin
OR: Odds ratio
CI: Confidence intervals
WHO: The World Health Organization

## References

[1] Jackson SJ, Steer AC, Campbell H (2011). Systematic review: estimation of global burden of non-suppurative sequelae of upper respiratory tract infection: rheumatic fever and post-streptococcal glomerulonephritis. Trop Med Int Health.

[2] Seckeler MD, Hoke TR (2011). The worldwide epidemiology of acute rheumatic fever and rheumatic heart disease. Clin Epidemiol.

[3] James SL, Abate D, Abate KH, Abay SM, Abbafati C, Abbasi N (2018). Global, regional, and national incidence, prevalence, and years lived with disability for 354 diseases and injuries for 195 countries and territories, 1990–2017: a systematic analysis for the Global Burden of Disease Study 2017. Lancet.

[4] Yadeta D, Hailu A, Haileamlak A, Gedlu E, Guteta S, Tefera E (2016). Prevalence of rheumatic heart disease among school children in Ethiopia: a multisite echocardiography-based screening. Int J Cardiol.

[5] Gemechu T, Mahmoud H, Parry EH, Phillips DI, Yacoub MH (2017). Community-based prevalence study of rheumatic heart disease in rural Ethiopia. Eur J Prev Cardiol.

[6] Tadele H, Mekonnen W, Tefera E (2013). Rheumatic mitral stenosis in children: more accelerated course in sub-saharan patients. BMC Cardiovasc Disord.

[7] Steer AC, Colquhoun S, Kado J, Carapetis JR (2011). Secondary prophylaxis is important for the prevention of recurrent rheumatic fever in the Pacific. Pediatr Cardiol.

[8] Penicillin for secondary prevention of rheumatic fever-Manyemba, J-2002 | Cochrane Library [Internet]. [cited 2020 Dec 1]. Available from: 10.1002/14651858.CD002227/abstract 10.1002/14651858.CD002227 PMC7017848 12137650

[9] Steer AC, Carapetis JR (2009). Prevention and treatment of rheumatic heart disease in the developing world. Nat Rev Cardiol.

[10] Karki P, Uranw S, Bastola S, Mahato R, Shrestha NR, Sherpa K, et al. Effectiveness of Systematic Echocardiographic Screening for Rheumatic Heart Disease in Nepalese Schoolchildren: A Cluster Randomized Clinical Trial. JAMA Cardiol [Internet]. 2021 Jan 20 [cited 2021 Feb 18]; Available from: https://jamanetwork.com/journals/jamacardiology/fullarticle/2775558 10.1001/jamacardio.2020.7050 PMC7818193 33471029

[11] de Dassel JL, de Klerk N, Carapetis JR, Ralph AP (2018). How many doses make a difference? an analysis of secondary prevention of rheumatic fever and rheumatic heart disease. J Am Heart Assoc.

[12] Gerber MA, Baltimore RS, Eaton CB, Gewitz M, Rowley AH, Shulman ST (2009). Prevention of rheumatic fever and diagnosis and treatment of acute streptococcal pharyngitis. Circulation.

[13] Organization WH. Rheumatic fever and rheumatic heart disease: report of a WHO expert consultation, Geneva, 29 October–1 November, 2001. World Health Organization; 2004.

[14] Mekonen KK, Yismaw MB, Abiye AA, Tadesse TA (2020). Adherence to benzathine penicillin G secondary prophylaxis and its determinants in patients with rheumatic heart disease at a cardiac center of an Ethiopian tertiary care teaching hospital. Patient Prefer Adherence.

[15] Adem A, Dukessa Gemechu T, Jarso H, Reta W (2020). Rheumatic heart disease patients’ adherence to secondary prophylaxis and associated factors at hospitals in jimma zone, Southwest Ethiopia: a multicenter study. Patient Prefer Adherence.

[16] Aa A, Gn G. Assessment of rheumatic heart disease adherence to secondary prophylaxis and factor affecting among patients with rheumatic heart disease attending Jimma University Teaching Hospital, South West of Ethiopia. International Journal of Cardiovascular Research [Internet]. 2019 Apr 29 [cited 2021 Feb 18];2019. Available from: https://www.lendongku.com/peer-review/assessment-of-rheumatic-heart-disease-adherence-to-secondary-prophylaxis-and-factor-affecting-among-patients-with-rheumatic-heart--UtC5.pdf.

[17] Oli K, Porteous J (1999). Rheumatic heart disease among school children in Addis Ababa City: awareness and adequacy of its prophylaxis. Ethiop Med J.

[18] Bariso M, Adi W (2019). Adherence of rheumatic heart disease patients to secondary prophylaxis and main reasons for poor adherence at Jimma Medical Center. E J Cardiovasc Med.

[19] Wilson N (2010). Rheumatic heart disease in indigenous populations—New Zealand experience. Heart Lung Circ.

[20] Mincham CM, Toussaint S, Mak DB, Plant AJ (2003). Patient views on the management of rheumatic fever and rheumatic heart disease in the kimberley: a qualitative study. Aust J Rural Health.

[21] Wyber R, Kado J, Dougherty S, Carapetis J, Zühlke L, Wilson N (2021). Chapter 12-rheumatic heart disease control programs, registers, and access to care. Acute rheumatic fever and rheumatic heart disease.

[22] Gasse B, Baroux N, Rouchon B, Meunier J-M, Frémicourt ID, D’Ortenzio E (2013). Determinants of poor adherence to secondary antibiotic prophylaxis for rheumatic fever recurrence on Lifou, New Caledonia: a retrospective cohort study. BMC Public Health.

[23] Amarilyo G, Chodick G, Zalcman J, Koren G, Levinsky Y, Somekh I (2019). Poor long-term adherence to secondary penicillin prophylaxis in children with history of rheumatic fever. Semin Arthritis Rheum.

[24] Clinical Outcomes in 3343 Children and adults with rheumatic heart disease from 14 low- and middle-income countries | circulation [Internet]. [cited 2021 Feb 22]. Available from: 10.1161/CIRCULATIONAHA.116.024769. 10.1161/CIRCULATIONAHA.116.024769 27702773

[25] Rémond MGW, Coyle ME, Mills JE, Maguire GP (2016). Approaches to improving adherence to secondary prophylaxis for rheumatic fever and rheumatic heart disease: a literature review with a global perspective. Cardiol Rev.

[26] Zühlke L, Karthikeyan G, Engel ME, Rangarajan S, Mackie P, Cupido-Katya Mauff B (2016). Clinical outcomes in 3343 children and adults with rheumatic heart disease from 14 low- and middle-income countries: two-year follow-up of the global rheumatic heart disease registry (the REMEDY Study). Circulation.

[27] Global Unmet Needs in Cardiac Surgery - ScienceDirect [Internet]. [cited 2021 Feb 22]. Available from: https://www.sciencedirect.com/science/article/pii/S2211816018300942.

[28] Yacoub M, ElGuindy A, Afifi A, Yacoub L, Wright G (2014). Taking cardiac surgery to the people. J Cardiovasc Trans Res.

[29] Abrams J, Watkins DA, Abdullahi LH, Zühlke LJ, Engel ME (2020). Integrating the prevention and control of rheumatic heart disease into country health systems: a systematic review and meta-analysis. Glob Heart.

[30] Predictors of Stunting among School-Age Children in Northwestern Ethiopia [Internet]. [cited 2021 Dec 8]. Available from: https://www.hindawi.com/journals/jnme/2018/7521751/. 10.1155/2018/7521751 PMC6171210 30327729

[31] Beaton A, Zühlke L, Mwangi J, Taubert KA (2020). Rheumatic heart disease and COVID-19. Eur Heart J.

