# Supplementary material for: Secondary prevention of rheumatic heart disease in Ethiopia: a multicenter study
Source: BMC Cardiovasc Disord. 2022 Feb 2;22:26. doi: 10.1186/s12872-022-02473-4 (PMC8809239; doi:10.1186/s12872-022-02473-4)
Supplement: Supplementary file 1 — Additional file 1. Data collection tool is available as a supplemental file. [file 12872_2022_2473_MOESM1_ESM.pdf]

Demographic data

Study ID	________________________
Gender	◯ Female<br>◯ Male
Current Age (years)	________________________
Age at diagnosis of RHD	________________________
Family size (number of people living in the same house as the child) if known	________________________
Place of residence	◯ Rural<br>◯ Urban
Address (Zone, Woreda, Kebele of town)	________________________
Estimated distance from the nearest Cardiology Clinic (kms)	________________________
Level of education of parents	◯ Not educated at all<br>◯ Primary school completed<br>◯ High school graduate<br>◯ College graduate<br>◯ Uknown
Occupation of parents/Guardians	◯ Gov't institution<br>◯ Private company<br>◯ Farmers<br>◯ Others (Small scale business, service delivery)
Estimated monthly income (in birr) of the family	________________________
Frequency of cardiac clinic follow up (months interval)	________________________

Clinical data

Study ID	__________________________________
Duration of Illness at diagnosis (in months)	__________________________________
Presentation at diagnosis	☐ Fever<br>☐ Arthritis<br>☐ Arthralgia<br>☐ Sydenham's Chorea<br>☐ Subcutaneous nodules<br>☐ Erythema marginatum<br>☐ ECHO evidence of Cardiac involvement<br>☐ Elevated inflammatory markers (ESR, CRP, ASO)<br>☐ Abnormal EKG<br>☐ Others<br>☐ None
Current symptoms	☐ Shortness of breath<br>☐ Chest pain<br>☐ Palpitations<br>☐ Dizziness<br>☐ Syncope<br>☐ None
Cardiac involvement	◯ Yes<br>◯ No
Type of Valvular lesion	☐ Mitral valve disease<br>☐ Aortic valve disease<br>☐ Tricuspid valve disease<br>☐ Pulmonary valve disease<br>☐ Others
Mitral valve disease	◯ Trivial to Mild<br>◯ Moderate<br>◯ Severe<br>◯ None
Aortic valve disease	◯ Trivial to Mild<br>◯ Moderate<br>◯ Severe<br>◯ None
History of recurrence of RF since diagnosis	◯ Yes<br>◯ No<br>◯ Unknown
Number of times of recurrence	__________________________________

Medications other than Prophylaxis	☐ Lasix<br>☐ Spironolactone<br>☐ Digoxin<br>☐ Other diuretics (Specify)<br>☐ Anticoagulants<br>☐ Antiplatelet<br>☐ None
History of cardiac surgery	◯ Yes<br>◯ No
History of Percutaneous Interventions	◯ Yes<br>◯ No

Prophylaxis data

Study ID	__________________________________
Patient currently on secondary Prophylaxis for RHD	◯ Yes<br>◯ No
Type of secondary prophylaxis	◯ Benzathine Penicillin<br>◯ Oral Penicillin<br>◯ Amoxicillin<br>◯ Other medications (specify)
Duration of prophylaxis (since initiation) in months	__________________________________
Were there missed prophylaxis in the last 12 months?	◯ Yes<br>◯ No
How many prophylactic doses are missed over the past 12 months on average? (for IM Benzathine Penicillin users)	__________________________________
How many prophylactic doses are missed over the past 12 months on average? (for PO penicillin and Amoxicillin users)	__________________________________
Reason for missed doses/not taking prophylaxis (If any)	☐ Didn't remember<br>☐ Run out of Medication<br>☐ Fear of pain during injection<br>☐ Live too far to come to the clinic<br>☐ Cannot afford the medicine<br>☐ Other reasons (specify)
Othe reasons for missed prophylaxis doses	__________________________________
Allergy to Prophylaxis	◯ Yes<br>◯ No<br>◯ Unknown
Prophylactic medication availability	◯ Always available<br>◯ Sometimes<br>◯ Easy to find<br>◯ Not available most of the time
Is the family paying for the prophylaxis medication?	◯ Yes<br>◯ No
Perception about the effectiveness of secondary prophylaxis	◯ Beneficial<br>◯ Not beneficial<br>◯ Not sure

Perception of clinical course of the patient	◯ Improving<br>◯ Not improving<br>◯ Same<br>◯ Not sure<br>◯ Others (Specify)
